# T Cells in Chronic Lymphocytic Leukemia: A Two-Edged Sword

**DOI:** 10.3389/fimmu.2020.612244

**Published:** 2021-01-20

**Authors:** Elisavet Vlachonikola, Kostas Stamatopoulos, Anastasia Chatzidimitriou

**Affiliations:** ^1^Centre for Research and Technology Hellas, Institute of Applied Biosciences, Thessaloniki, Greece; ^2^Department of Genetics and Molecular Biology, Faculty of Biology, Aristotle University of Thessaloniki, Thessaloniki, Greece; ^3^Department of Molecular Medicine and Surgery, Karolinska Institutet, Stockholm, Sweden

**Keywords:** chronic lymphocytic leukemia, T lymphocytes, anti-tumor immunity, tumor microenvironment, T cell-based therapies

## Abstract

Chronic lymphocytic leukemia (CLL) is a malignancy of mature, antigen-experienced B lymphocytes. Despite great progress recently achieved in the management of CLL, the disease remains incurable, underscoring the need for further investigation into the underlying pathophysiology. Microenvironmental crosstalk has an established role in CLL pathogenesis and progression. Indeed, the malignant CLL cells are strongly dependent on interactions with other immune and non-immune cell populations that shape a highly orchestrated network, the tumor microenvironment (TME). The composition of the TME, as well as the bidirectional interactions between the malignant clone and the microenvironmental elements have been linked to disease heterogeneity. Mounting evidence implicates T cells present in the TME in the natural history of the CLL as well as in the establishment of certain CLL hallmarks e.g. tumor evasion and immune suppression. CLL is characterized by restrictions in the T cell receptor gene repertoire, T cell oligoclonal expansions, as well as shared T cell receptor clonotypes amongst patients, strongly alluding to selection by restricted antigenic elements of as yet undisclosed identity. Further, the T cells in CLL exhibit a distinctive phenotype with features of “exhaustion” likely as a result of chronic antigenic stimulation. This might be relevant to the fact that, despite increased numbers of oligoclonal T cells in the periphery, these cells are incapable of mounting effective anti-tumor immune responses, a feature perhaps also linked with the elevated numbers of T regulatory subpopulations. Alterations of T cell gene expression profile are associated with defects in both the cytoskeleton and immune synapse formation, and are generally induced by direct contact with the malignant clone. That said, these abnormalities appear to be reversible, which is why therapies targeting the T cell compartment represent a reasonable therapeutic option in CLL. Indeed, novel strategies, including CAR T cell immunotherapy, immune checkpoint blockade and immunomodulation, have come to the spotlight in an attempt to restore the functionality of T cells and enhance targeted cytotoxic activity against the malignant clone.

## Introduction

Chronic lymphocytic leukemia (CLL) is an age-related malignancy characterized by the accumulation of monoclonal mature B cells expressing CD5, CD19 and CD23 on their surface (1). Numerous studies support that the clonotypic B cell receptor immunoglobulin (BcR IG) is critically implicated in disease pathophysiology (2). Indeed, the intensity of intracellular signaling downstream of the BcR in CLL cells is associated with cell proliferation and disease severity (3), whereas restrictions in the gene repertoire of the clonotypic BcR IG strongly highlight the role of antigenic triggering in disease pathogenesis (4, 5).

Different lines of evidence suggest that interactions with two main categories of microenvironmental components i.e. soluble factors and other extracellular elements as well as bystander cells are implicated in shaping a supportive tumor microenvironment (TME) in CLL (6–8). The former category includes various extracellular molecules (e.g.) cytokines, chemokines, antigens but also, in a broader sense, products of metabolic processes. Indeed, although cellular metabolism in CLL remains largely unexplored, increasing evidence implicates oxidative stress in the natural history of CLL by showing e.g. that the accumulation of reactive oxygen species (ROS) may hold prognostic significance (9, 10).

Turning to bystander cells, different subpopulations of T cells as well as natural killer (NK) cells accumulate *in vivo* along with mesenchymal stromal cells (MSC) and nurse-like cells (NLCs), forming a complex network that favors clonal expansion and proliferation of the malignant clone (11–13). Ongoing crosstalk of CLL malignant cells with these other cell populations in the TME affects the function of both parties. On the one hand, this leads to immunosuppression, a hallmark of CLL associated with increased susceptibility to infections, autoimmune manifestations, and a higher incidence of secondary malignancies (14). On the other hand, external triggers support the survival and proliferation of the neoplastic cells (15); this was first made evident when it was found that CLL cells undergo apoptosis in suspension cultures, which can be partially rescued by co-cultures with stromal cells or NLC (11).

T cells are major contributors to adaptive immunity, actively engaged in defense against pathogens and tumor cells through a great variety of accessory and effector functions. Upon encounter with a specific antigen, T cells are activated and eventually differentiate into various distinct subpopulations, acquiring either cytotoxic or helper properties. Pathogen clearance, mediated by cytotoxic T cells or through the activation of other cell types induced by cytokines secreted from T helper cells, is followed by the apoptosis of the effector T cells as a homeostatic mechanism that restores the immune system at the pre-activation state. Simultaneously, a small fraction of antigen-specific memory T cells are resting in the body, ready to generate an immediate and effective secondary response (16, 17). This homeostatic balance is perturbed in CLL, where, similar to various solid or hematological malignancies, T cells exhibit a number of phenotypic and functional defects undermining their normal immune responses (18). Moreover, T cells appear to have an active involvement in CLL development and evolution, as supported by experimental evidence that the transfer of autologous activated T cells in NOD/Shi-scid, γcnull (NSG) mice is a prerequisite for successful engraftment of CLL cells in murine models (19, 20). Interestingly, the post-transfer outgrowth of functionally competent Th1 T cells seen in NSG mice highlights the suppressive and inhibitory TME in CLL patients, particularly considering reports that these T cells can regain their functionality and promote B cell diversification and differentiation (18). It has been proposed that this phenomenon may reflect selection for Th1 cells *in vivo*, release from various inhibitory mechanisms operating within the human host or a special effect of the distinct NSG microenvironment. Arguably, similar mechanisms may also act in CLL patients, however, strong correlations remain elusive (20), highlighting the need for caution when attempting extrapolations based on findings deriving from model systems.

In CLL, as in other cancers, genomic instability leads to various alterations that serve as a source of cognate antigens, sufficient for the induction of tumor-reactive T cell responses (21–24). Although tumor-/antigen-specific T cells have been identified in CLL patients, these are incapable of effectively eliminating neoplastic cells due to several intertwined immunosuppressive mechanisms that establish T cell dysfunction (25, 26), thus, favoring escape from immune surveillance (27). Evidence suggests that this tolerogenic TME is actively induced by the CLL cells themselves through the secretion of cytokines and chemokines that affect cellular functions in the bystander cells (15). As a matter of fact, a recent study demonstrated that shared peptides from the highly conserved BcR IG of patients belonging to CLL stereotyped subsets 1 and 2 could generate antigen-specific T cells upon presentation in HLA-restricted manner. Furthermore, that study reported T cell specific reactivity against CLL cells, while also showing that immunization of Eμ-TCL1 mice with BcR IG-derived peptides led to anti-leukemic T cell responses, implying that targeted T cell immunotherapy against the clonotypic BcR IG represents an appealing treatment option (28). Along similar lines, the identification of the unique set of neo-epitopes of each patient could lead to the isolation of leukemia-specific T cells, that can be used as a novel treatment approach (29).

Taken together, deciphering the cross-talk mechanisms between CLL cells and bystander T cells as well as the implicated signaling pathways is reasonably anticipated to offer insights into disease pathophysiology, and, potentially, a great opportunity for designing microenvironment-directed treatment approaches.

## T Cell Subpopulation Imbalances in CLL

Alterations in the T cell composition have been extensively described in CLL, however the exact impact of these changes on disease development and progression remains controversial. The first described T cell abnormality in a CLL-specific context concerned the finding of increased numbers of T cells in the periphery, accounting mostly for CD8^+^ T cell expansion that leads to inversion of the normal CD4^+^/CD8^+^ cell ratio (30). Of note, these CD8^+^ T cells are severely compromised as to their capacity to exert cytotoxicity (31). Regarding the potential clinical relevance of these findings, it has been reported that elevated numbers of CD8^+^ cells are associated with disease progression as well as shorter time to first treatment and progression-free survival, perhaps partly due to co-expression of the inhibitory receptor of programmed death-1 (PD-1) (32). However, under different conditions oligoclonal expansion of CD8^+^ effector cells in the Eµ-TCL1 mouse model of CLL has been associated with disease control, whereas conversely, ablation of CD4^+^ T cells did not affect disease progression (33).

Expansions of different CD4^+^ T cell subpopulations that exert either pro-tumoral activity or immunosuppression have been reported in CLL. Early studies have shown that IFN-γ secreting Th1 cells can provide trophic signal for CLL cells inhibiting programmed cell death and supporting survival (34). On the other hand, T cell-mediated immune responses have been linked with disease control in cases of autologous CLL regression, highlighting the multi-dimensional T cell activity into the CLL TME (35). Further, expansion of a novel CD4^+^ T subpopulation characterized by the expression of the TIGIT (T cell immunoreceptor with Ig and ITIM domains) inhibitory receptor has been recently observed in patients at advanced stages of CLL; these cells were shown to support CLL survival in *in vitro* experiments (36). Finally, CD4^+^PD-1^+^HLA-DR^+^ T cells that co-express inhibitory and activation markers have been associated with aggressive disease (37). Altogether, these apparently conflicting findings clearly indicate the need for delving deeper into the distinct subsets and functions of the T cell compartment in CLL.

A well-characterized finding in CLL concerns the elevated numbers of T regulatory cells (Tregs) (30, 38) that are generally known to contribute to cancer progression through dampened antitumor responses and immunosuppression (39, 40). Of note, CLL Tregs are more suppressive than normal Tregs, whereas depletion of these cells led to efficient anti-tumor responses in animal models of CLL (41, 42). Additionally, interleukin 4 (IL-4) secreted from Tregs also induces anti-apoptotic pathways in CLL cells through the overexpression of the anti-apoptotic protein BCL2, which is therapeutically targeted by venetoclax, an effective agent for the treatment of CLL (43–46). Hence, efforts to inhibit Tregs through targeting the FoxP3 transcription factor, that is critical for their function (47, 48), or other interacting molecules in the downstream pathway could be clinically useful, at least in principle, similar to what has been proposed for other cancers (49, 50).

Th17 cells represent another T cell subpopulation with a critical role in immune homeostasis, actively participating in inflammatory processes. Imbalances in Th17 populations have been linked with autoimmune disorders, however their role in cancer remains to be fully elucidated (51). In CLL, Th17 cells are increased compared to healthy individuals; however, increased Th17 cells in cases at early disease stages have been reported to be associated with a favorable clinical outcome, conceivably through controlling the expansion of Tregs (52, 53). Altogether, the aforementioned evidence suggests that the balance between Tregs and Th17 cells may impact on the clinical outcome through as yet unknown mechanisms (14, 51).

## Functional Impairment of T Cells in CLL: Causes, Functional Consequences, and Clinical Implications

T cell activation starts when an antigen-presenting cell (APC) introduces an epitope bound on an HLA molecule to a specific TR. A second signal from the CD28 receptor, constitutively expressed on T cells, enhances cell proliferation, generation of cytotoxic lymphocytes, and cytokine production (54). In CLL, *in vitro* T cell activation through CD28 followed by infusion of the activated autologous T cells to the patients was shown to lead to competent anti-leukemic effects and decreased TR clonality. Based on this evidence, *ex vivo* T cell activation and expansion was proposed as a valid treatment option in CLL, however failed to gain ground (55).

Effective activation of naïve T cells through normal interactions with APCs requires the formation of the immune synapse, a specialized contact area between T cells and APCs (56). Impaired immune synapse formation has been documented as a hallmark of CLL and shown to be mechanistically linked to alterations in the expression of genes associated with actin polymerization, cytoskeletal organization and vesicle trafficking (57). Of note, immune synapse defects appear to be contact-dependent, as shown in both *ex vivo* studies of primary patient samples and *in vivo* studies of animal models of CLL (58, 59).

Such effects may dampen effective antitumor responses, through a complex network of inhibitory signals and immunosuppressive interactions amongst CLL cells and T cells into TME. Importantly, signals delivered through the CTLA-4 and PD-1 receptors modulate T cell activation status through reduction of TR signaling and cytolytic functions. These inhibitory molecules are overexpressed in T cells of patients with CLL, while their ligands are also overexpressed by the malignant cells, hence putting the brakes on anti-tumor activity (60). Specifically, CLL cells express inhibitory surface molecules from B7 and TNF-receptor families, namely CD200, CD274 (PD-L1), CD276 and CD270 which are implicated in synapse formation defects in both autologous and allogeneic T cells, suggesting a novel evasion mechanism (61).

On these grounds, research into these receptors and ligands could pave the way to novel treatment modalities for CLL, whereby T cell-driven immune responses could be re-invigorated through targeting the interactions between inhibitory receptors and their ligands. However, despite encouraging pre-clinical results, the clinical application of antibodies against these receptors (immune checkpoint inhibitors [ICI]) has been frustratingly disappointing in CLL, with only minimal effectiveness when administered as monotherapy (62, 63). Evidently, more research is warranted in order to define the precise role of ICIs in the management of CLL and also better understand the mechanisms underlying the emergence of severe adverse events reported for these agents (64, 65).

In CLL, immune synapse defects were found to be reversible by the administration of lenalidomide, an immunomodulatory drug (IMiD) that appears to reshape the expression of cytokines and orchestrate cell-mediated responses, mobilizing the T cell compartment (66, 67). In addition, lenalidomide treatment invigorates T cell motility and migration through activation of integrin lymphocyte function–associated antigen-1 (LFA-1), also affected by direct contact with CLL cells (68). Relevant to mention, clinical studies of lenalidomide as monotherapy or consolidation therapy after chemoimmunotherapy have reported restored immune synapse formation and downstream signaling and well as improved quality of clinical responses (69, 70).

T cells in CLL display features of exhaustion, a functional state initially described in the setting of chronic infections but later also recognized in various cancer types (25, 71–75). In such conditions, exhaustion has been postulated to arise as a result of persistent antigenic stimulation, leading to gradual loss of T cell effector functions (76–78). Similar to other contexts, exhausted T cells in CLL are characterized by low proliferative rates, reduced cytotoxicity, altered cytokine production and, as already mentioned, expression of multiple inhibitory receptors on their surfaces, including CTLA-4, PD-1 and LAG-3 (25, 79, 80).

**Figure 1:**
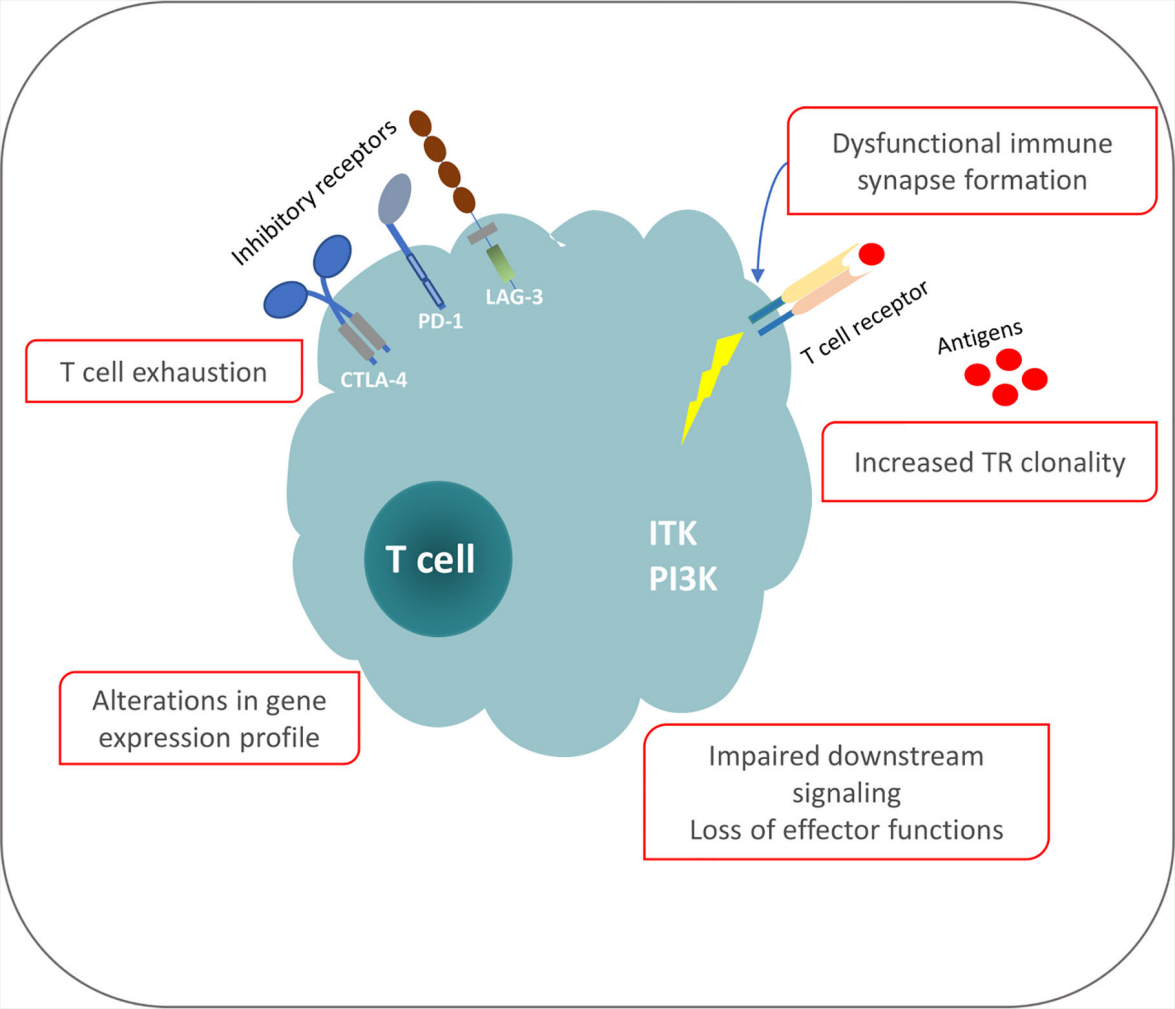
Phenotypic and functional defects of T cells in CLL.

The functional analogies between T cells in individuals exposed to persistent antigenic stimulation due to a chronic infection and individuals affected by CLL allows arguing that antigenic pressure is key to shaping the T cell repertoire and functionality also in CLL. That notwithstanding, the exact nature of the cognate antigens/epitopes implicated in T cell selection in CLL is yet to be defined. However, the most prominent (classes of) candidates concern: (i) the same antigens that are implicated in the selection of the CLL progenitors or the malignant cells themselves; (ii) tumor-derived epitopes from aberrant proteins expressed by the malignant cells e.g. due to a pathogenic mutation or other genomic aberration; and (iii) the clonotypic IG, perhaps the most abundant clone-specific molecule (81–84) (**Figure 1**).

## Antigen Selection Shapes the T Cell Compartment in CLL: Immunogenetic Evidence

The clonotypic BcR IG is critically implicated in the natural history of CLL, a fact amply supported by the therapeutic efficacy of agents interfering with BcR IG signaling, which have changed dramatically the therapeutic landscape of CLL (85–87). This clinical evidence complements immunogenetic evidence that the BcR IG is a key driver in disease ontogeny and evolution. In more detail, the BcR IG gene repertoire is characterized by remarkable restrictions (88), culminating in BcR IG stereotypy where, at clear odds with serendipity, different clones share (quasi)identical IG (4, 5, 89). Moreover, the molecular characteristics of the clonotypic BcR IG have established prognostic and predictive significance since patients with a significant imprint of somatic hypermutation (SHM) display indolent disease, in contrast to those with few or no SHM who generally follow a more aggressive clinical course (90, 91). Taken together, these features emphasize the links between BcR IG structure and clonal behavior, while also highlighting the importance of antigen selection in CLL pathophysiology (92, 93).

Considering the importance of extracellular cues for CLL cell survival and proliferation, as well the T cell compartment defects in CLL patients, the interest in the TR gene repertoire of T cells in CLL and the implicated antigens is hardly surprising. Immunophenotypic studies in the 1990s described T cell clonal expansions in CLL patients, prompting speculations that these cells could be possibly related to the host immune response against CLL-derived antigens, or emerged as a result of a longitudinal interplay between CLL and T cells in the proliferative centers (94). Moreover, analyses of the TR beta (TRB) chain gene rearrangements by Southern blotting offered the first molecular evidence for skewed TRBV gene repertoire and oligoclonality in both CD4^+^ and CD8^+^ T cells of CLL patients compared to healthy aged-matched controls (95–97). Additionally, stimulation of isolated T cells from CLL patients with autologous malignant cells resulted in monoclonal TR expansions, prompting speculations that CLL patients could bear a pool of T cells that specifically recognize leukemia-associated antigens (95, 98). Similar findings of decreased clonal diversity were also reported in Eμ-TCL1 mice supporting the notion of CLL-dependent antigen-driven selection pressure on the TR gene repertoire (99).

These initial studies were followed by significantly more extended examinations of the TR gene repertoire by our group, first through subcloning of TRBV-TRBD-TRBJ gene rearrangements followed by Sanger sequencing (100) and, more recently, by high-throughput approaches using next generation sequencing (NGS) (101, 102). Summarizing our findings: (i) the TRBV gene repertoire is skewed; (ii) oligoclonal T cell expansions feature prominently, particularly amongst cytotoxic T cells; (iii) such expansions persist and may even increase during the disease course; and, (iv) identical or highly similar TR clonotypes were shared between different patients, especially those belonging to the same stereotyped subset, and these appeared to be CLL-biased as they were not present in other contexts. Altogether, these findings allow arguing that antigen selection has a key role in shaping the TR repertoire in CLL, while also favoring the intriguing hypothesis that the relevant antigens are most likely CLL-related (101). However, the exact mechanism that underlies T cell clonality and retains these expanded populations during the disease course remains elusive.

In order to further understand T cell compartment dynamics in CLL, we extended our studies to longitudinal investigation (pre/post-treatment) of patients treated with chemoimmunotherapy with the fludarabine-cyclophosphamide-rituximab (FCR) regimen or the BcR signaling inhibitors (BcRi) ibrutinib (IB) and idelalisib (the latter in combination with rituximab, R-ID). We found that T-cell clonality significantly increased at (i) 3 months in the FCR and R-ID treatment groups, and (ii) over deepening clinical response in the R-ID group, with a similar trend detected in the IB group. Perhaps more importantly, in contrast to FCR that induced T-cell repertoire reconstitution, BcRi retained pretreatment clones. Extensive comparisons of the CLL dataset against external TR sequence databases showed little similarity with other entities, but instead revealed major TR clonotypes shared exclusively by patients with CLL, supporting selection by conserved CLL-associated antigens. In addition, we assessed the functional impact of these treatments on T cells and found that (i) R-ID upregulated the expression of activation markers in effector memory T cells, and (ii) both BcRi improved antitumor T-cell immune synapse formation, in marked contrast to FCR (102). Here, it is worth mentioning the contradictory findings of an another NGS-based longitudinal study of the TR gene repertoire in IB-treated patients, where this therapy resulted in TR repertoire diversification (103). However, major differences in the experimental and analytical procedures render these two studies incomparable, highlighting the need for further harmonization of the NGS protocols and bioinformatics workflows. A limitation inherent to both studies concerns the fact that total CD3^+^ T cells rather than particular T cell subpopulations were investigated, which could lead to misinterpretation due to a “dilution effect” by bulk T cell analysis. Finally, robust conclusions regarding the links, if any, between T cell dynamics overtime and clinical outcome are still not possible: hence studies on larger, well-characterized cohorts remain of paramount importance.

Considering mounting evidence that the TR gene repertoire in CLL is antigen selected, identifying the cognate antigens is an obvious line of research, not least because it would assist in designing effective antigen-specific immunotherapy. Studies of HLA-presented antigenome and computational models for tumor-associated antigen prediction proposed the existence of disease specific antigens, deriving from CLL-related genomic aberrations, that were identified exclusively on neoplastic cells of CLL patients (104–106). Additionally, the broad representation of BcR IG-derived epitopes in patients across different disease (Binet) stages and through various treatments suggest them as potential candidates for peptide vaccines and objectives of neoantigen-targeting immunotherapies. However, immune tolerance mechanisms can turn the BcR IG-derived peptides, a potentially significant pool of cognate neo-antigens in B cell lymphomas (107, 108), into ineffective targets for T cells. This phenomenon, known as immunoediting, has been described in different contexts (including cancer and viral infections) and can neutralize an immunogenic phenotype favoring cells to survive and proliferate (27, 109).

## Treatment Options in CLL and Implication of the T Cell Compartment

T cells in the CLL TME exhibit a number of phenotypic and functional defects, acquired through persistent crosstalk with the neoplastic cells. The T cell compartment aberrations appear to be CLL-specific and induced by the CLL cells, leading to functional exhaustion, reduced activation and proliferation of effector subpopulations and decreased cytotoxic responses. Thus, CLL cells have the ability to amend alter T cell functions in order to evade immune-surveillance, proliferate and further expand overtime.

**Figure 2:**
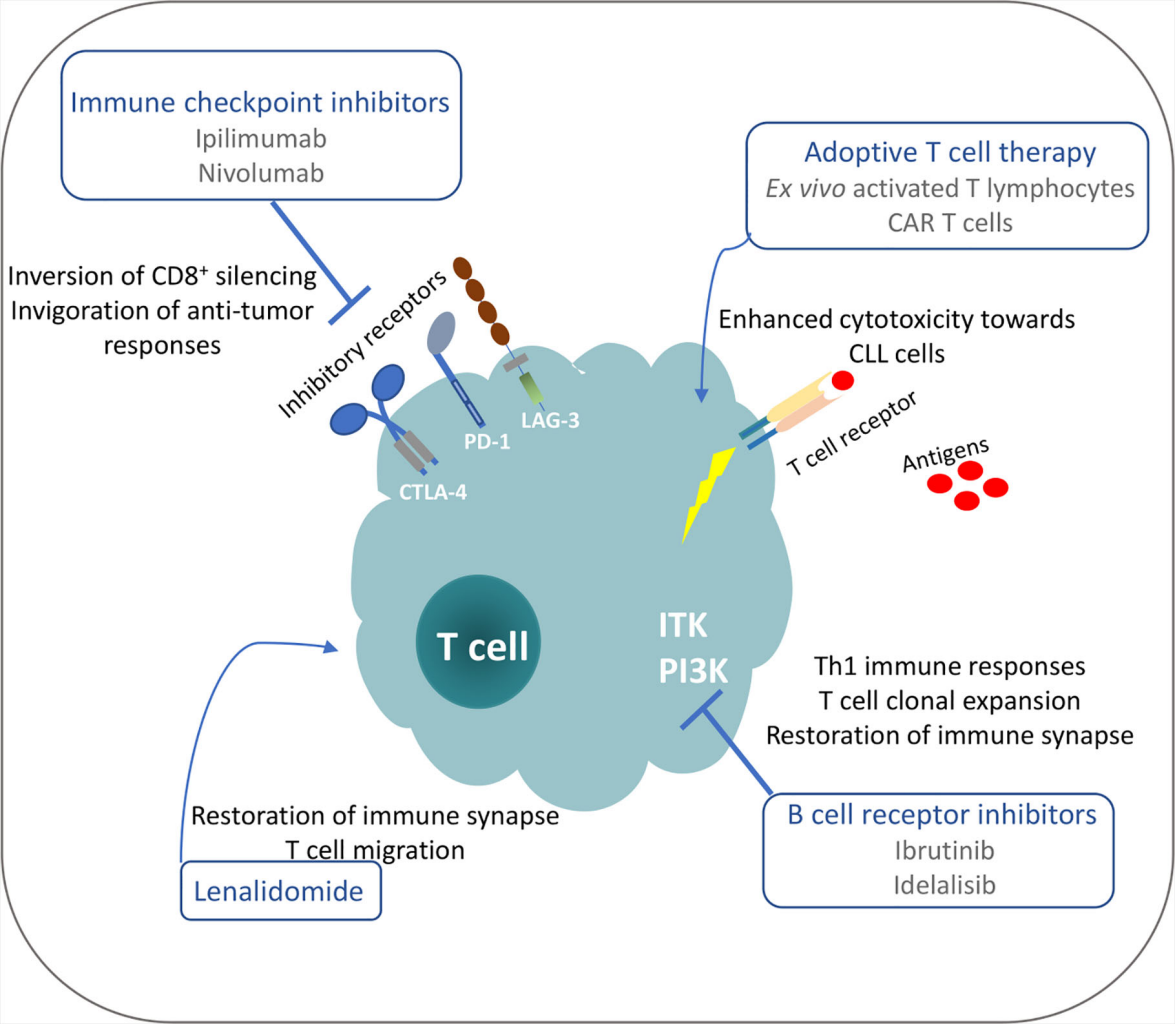
Possible mechanisms for restoration of T cell functionality in CLL under different treatment manipulations.

Despite the severe T cell deficiencies identified in CLL patients and models, studies demonstrate that subsets of these defective T cells can restore their functionality and mount anti-tumor responses under particular therapeutic interventions (**Figure 2**). Moreover, the identification of tumor-derived antigens implicated in T cell clonal expansions that may harbor anti-tumor properties can pave the way for therapeutic peptide vaccination strategies and adoptive T-cell transfer protocols in order to augment efficacy and guide the specificities of anticancer immune responses. Consequently, deep understanding of tumor-derived T cell dysfunction is highly significant for the development of new therapeutic modalities to restore anti-tumor immunity and result in tumor control.

In recent years, the advent of BCRi has led to a major paradigm change in the treatment of CLL. Due to structural and functional similarities of kinases between T and B cells, cross-reactivity of these agents has been described, highlighting an additional immunomodulatory mechanism of action in TME. In more detail, besides inhibiting the Bruton’s tyrosine kinase (BTK) that is implicated in signal transduction of the activated BcR in CLL cells (110), ibrutinib targets also the T-cell associated kinase ITK, modulating T cell immunity. This is made manifest by effective immune synapse formation, shift towards Th1 polarization, and expansion of functionally competent specific-T clones, possibly contributing in deepening clinical response (102, 111, 112). In addition, ibrutinib-induced down-regulation of the PD-1/PD-L1 axis and inhibition of the STAT3 pathway modulates the immunosuppressive TME: on these grounds, combination protocols with lenalidomide or checkpoint inhibitors hold promises for reactivation of anti-tumor immunity (113–115). Similar effects have been demonstrated by treatment with idelalisib that also targets other PI3K isoforms in T cells, enhancing anti-tumor functions through modulation of Tregs (74). This is unsurprising considering that intracellular signaling that leads to suppression of T cell effector functions is extensively dependent on pathways implicating an isoform of PI3Kδ that constitutes an excellent target for idelalisib. At a first glance, this effect might be considered as an alternative mechanism to reduce immunosuppression and invigorate T cell responses; however, the described severe autoimmune adverse effects cannot be overlooked (116). Effects on the CLL TME are not restricted to BCRi though, since treatment with the BCL2 inhibitor venetoclax has been associated with reduced numbers of PD-1^+^ T cells and expression of inflammatory cytokines, suggesting a mechanism of immune recovery (117). Collectively, thorough investigation of the possible immunomodulatory effects of novel agents in CLL is of great relevance for future development of new combination therapeutic strategies.

As already mentioned, interactions between inhibitory receptors and their ligands, including the PD-1/PD-L1 axis, enable CLL cells to evade T cell immunosurveillance, thus proposing a new area for therapeutic targeting. Preclinical studies in a murine CLL model showed that immune checkpoint inhibition of PD-1/PD-L1 led to prevention of immune dysfunction and leukemia development, hence offering a rationale for the clinical testing of these regiments (58). Despite the promising results of PD-1 blockade in clinical trials for metastatic melanoma (MM), non-small cell lung cancer (NSCLC) and renal cell cancer (RCC) (118), anti–PD-1 monotherapy benefits only a small fraction of CLL patients who developed Richter syndrome (64). Nevertheless, combination therapies with regimens targeting different inhibitory pathways may offer better outcomes (71, 119).

Adoptive T cell therapy is an evolving field that shows promises in recent trials. The protocol is based on the infusion of genetically modified T lymphocytes with a particular specificity for antigen recognition on the malignant cells. In CLL, the expression of the CD19 surface molecule is used as a selective B cell target and autologous cytotoxic T cells are modified *ex vivo* to express a chimeric antigen receptor (CAR) with affinity to CD19 of CLL cells. The engagement of a CAR-T cell with a CD19^+^ B cell leads to T cell activation in a MHC-independent manner, redirecting cytotoxic effects towards CLL cells (120). Encouraging results from adoptive T cell therapy in B acute lymphoblastic leukemia have paved the way for trials in CLL: initial results were deemed positive, but the overall efficiency of the approach was limited in patients with bulky lymph node involvement (121). Attempts to induce the anti-tumor properties and the efficiency of the CAR-T cells are based on, amongst others, modifications for constitutive expression of the CD40 ligand that lead to proliferation and Th1 immune responses, whereas also combination with PD-1 blockade has been used in order to bypass CAR-T exhaustion (121, 122).

A major challenge for effective CAR-T cell designs is to enhance and guide specificity towards the malignant cells. Thus, identification and in-depth characterization of T cell clones bearing anti-tumor properties emerge as highly relevant. In parallel, detailed studies on the HLA-lingadome highlight tumor-derived antigens that are exclusively expressed on the leukemic cells and represent possible effective targets for adoptive T cell therapy (107, 123). Additionally, the identification of anti-CLL T clones and the respective disease-specific cognate antigens offers a rationale for peptide vaccination strategies in order to induce anti-tumor responses, but also for target recognition for T-cell based immunotherapy. Indeed, spectrometric studies on the naturally presented HLA ligands in CLL identified a pool of antigens exclusively expressed in CLL patients, providing information regarding the immunogenicity of the malignant cells while also offering new treatment targets (107). However, clinical studies targeting neoepitopes expressed in the malignant cells demonstrate enhanced rejection of tumor cells, underling the safety profile of these approaches (124, 125).

## Concluding Remarks

CLL cells have the ability to transform the effector functions of the bystander T cells in the TME, thus rendering them a source of trophic signals for the survival and proliferation of the malignant clone. Moreover, alterations in the T cell transcriptome combined with functional exhaustion undermine cytotoxic responses against tumor cells, establishing tumor evasion and escape of CLL cells from immunosurveillance. That said, despite the observed defects in the T cell compartment, several studies have identified subsets of T cells bearing anti-tumor properties that can be unleashed under the proper stimulation. This binary, contradicting role of T lymphocytes in CLL TME supports the analogy with a two-edged sword. Based on the observations discussed in the current review, the mobilization of exhausted T cells and the invigoration of anti-tumor pathways could be translated to clinical efficiency, tumor control and durable remissions. However, in order for this objective to be met, detailed characterization of the implicated mechanisms at the molecular and cellular level is urgently warranted.

## Author Contributions

All authors contributed to the article and approved the submitted version. EV wrote the manuscript. KS and AC edited the text and gave final approval.

## Funding

This work is supported in part by the ERANET Transcan-2, Project Acronym: Novel, and the Hellenic Precision Medicine Network in Oncology.

## Conflict of Interest

KS and AC have received unrestricted grant support from Jannsen Pharmaceutica and Abbvie.

The remaining author declares that the research was conducted in the absence of any commercial or financial relationships that could be construed as a potential conflict of interest.

## References

[1] Kipps TJ Stevenson FK Wu CJ Croce CM Packham G Wierda WG Chronic lymphocytic leukaemia. Nat Rev Dis Prim (2017) 3:1–22. 10.1038/nrdp.2016.96 PMC5336551 28102226

[2] Burger JA Chiorazzi N B cell receptor signaling in chronic lymphocytic leukemia. Trends Immunol (2013) 34:592–601. 10.1016/j.it.2013.07.002 23928062 PMC3898793

[3] Chiorazzi N Stevenson FK Celebrating 20 Years of IGHV Mutation Analysis in CLL. HemaSphere (2020) 4:e334. 10.1097/hs9.0000000000000334 32382709 PMC7000474

[4] Agathangelidis A Chatzidimitriou A Gemenetzi K Giudicelli V Karypidou M Plevova K Higher-order connections between stereotyped subsets: Implications for improved patient classification in CLL. Blood (2020). 10.1182/blood.2020007039 PMC7976441 32992344

[5] Stamatopoulos K Belessi C Moreno C Boudjograh M Guida G Smilevska T Over 20% of patients with chronic lymphocytic leukemia carry stereotyped receptors: Pathogenetic implications and clinical correlations. Blood (2007) 109:259–70. 10.1182/blood-2006-03-012948 16985177

[6] Shain KH Dalton WS Tao J The tumor microenvironment shapes hallmarks of mature B-cell malignancies. Oncogene (2015) 34:4673–82. 10.1038/onc.2014.403 PMC4688902 25639873

[7] Ghia P Chiorazzi N Stamatopoulos K Microenvironmental influences in chronic lymphocytic leukaemia: The role of antigen stimulation. J Internal Med (J Intern Med) (2008) 549–62. 10.1111/j.1365-2796.2008.02030.x 19017179

[8] Herishanu Y Katz BZ Lipsky A Wiestner A Biology of Chronic Lymphocytic Leukemia in Different Microenvironments. Clinical and Therapeutic Implications. Hematol Oncol Clin North Am (2013) 27:173–206. 10.1016/j.hoc.2013.01.002 23561469 PMC3660068

[9] D’Arena G Vitale C Perbellini O Coscia M La Rocca F Ruggieri V Prognostic relevance of oxidative stress measurement in chronic lymphocytic leukaemia. Eur J Haematol (2017) 99:306–14. 10.1111/ejh.12918 28646624

[10] D’Arena G Seneca E Migliaccio I De Feo V Giudice A La Rocca F Oxidative stress in chronic lymphocytic leukemia: still a matter of debate. Leuk Lymphoma (2019) 60:867–75. 10.1080/10428194.2018.1509317 30234409

[11] Burger JA Gribben JG The microenvironment in chronic lymphocytic leukemia (CLL) and other B cell malignancies: Insight into disease biology and new targeted therapies. Semin Cancer Biol (2014) 24:71–81. 10.1016/j.semcancer.2013.08.011 24018164

[12] Plander M Seegers S Ugocsai P Diermeier-Daucher S Iványi J Schmitz G Different proliferative and survival capacity of CLL-cells in a newly established in vitro model for pseudofollicles. Leukemia (2009) 23:2118–28. 10.1038/leu.2009.145 19657365

[13] Ghamlouch H Ouled-Haddou H Damaj G Royer B Gubler B Marolleau J-P A Combination of Cytokines Rescues Highly Purified Leukemic CLL B-Cells from Spontaneous Apoptosis In Vitro. PLoS One (2013) 8:e60370. 10.1371/journal.pone.0060370 23555960 PMC3608602

[14] Long M Beckwith K Do P Mundy BL Gordon A Lehman AM Ibrutinib treatment improves T cell number and function in CLL patients. J Clin Invest (2017) 127:3052–64. 10.1172/JCI89756 PMC5531425 28714866

[15] van Attekum MHA Eldering E Kater AP Chronic lymphocytic leukemia cells are active participants in microenvironmental cross-talk. Haematologica (2017) 102:1469–76. 10.3324/haematol.2016.142679 PMC5685246 28775118

[16] Kaech SM Ahmed R Memory CD8+ T cell differentiation: Initial antigen encounter triggers a developmental program in naïve cells. Nat Immunol (2001) 2:415–22. 10.1038/87720 PMC3760150 11323695

[17] Bajénoff M Granjeaud S Guerder S The strategy of T cell antigen-presenting cell encounter in antigen-draining lymph nodes revealed by imaging of initial T cell activation. J Exp Med (2003) 198:715–24. 10.1084/jem.20030167 PMC2194192 12953093

[18] Lamble AJ Lind EF Targeting the immune microenvironment in acute myeloid leukemia: A focus on T cell immunity. Front Oncol (2018) 8:213. 10.3389/fonc.2018.00213 29951373 PMC6008423

[19] Bagnara D Kaufman MS Calissano C Marsilio S Patten PEM Simone R A novel adoptive transfer model of chronic lymphocytic leukemia suggests a key role for T lymphocytes in the disease. Blood (2011) 117:5463–72. 10.1182/blood-2010-12-324210 PMC3109718 21385850

[20] Patten PEM Ferrer G Chen S-S Simone R Marsilio S Yan X-J Chronic lymphocytic leukemia cells diversify and differentiate in vivo via a nonclassical Th1-dependent, Bcl-6–deficient process. JCI Insight (2016) 1(4):e86288. 10.1172/jci.insight.86288 27158669 PMC4855875

[21] Baliakas P Jeromin S Iskas M Puiggros A Plevova K Nguyen-Khac F Cytogenetic complexity in chronic lymphocytic leukemia: Definitions, associations, and clinical impact. Blood (2019) 133:1205–16. 10.1182/blood-2018-09-873083 PMC6509568 30602617

[22] Schumacher TN Schreiber RD Neoantigens in cancer immunotherapy. Science (80- ) (2015) 348:69–74. 10.1126/science.aaa4971 25838375

[23] Tausch E Mertens D Stilgenbauer S Genomic features: Impact on pathogenesis and treatment of chronic lymphocytic leukemia. Oncol Res Treat (2016) 39:34–40. 10.1159/000443906 26890126

[24] Gaidano G Rossi D The mutational landscape of chronic lymphocytic leukemia and its impact on prognosis and treatment. Hematology (2017) 2017:329–37. 10.1182/asheducation-2017.1.329 PMC6142556 29222275

[25] Riches JC Davies JK McClanahan F Fatah R Iqbal S Agrawal S T cells from CLLpatients exhibit features of T-cell exhaustion but retain capacity for cytokine production. Blood (2013) 121:1612–21. 10.1182/blood-2012-09-457531 PMC3587324 23247726

[26] Gitelson E Hammond C Mena J Lorenzo M Buckstein R Berinstein NL Chronic lymphocytic leukemia-reactive T cells during disease progression and after autologous tumor cell vaccines. Clin Cancer Res (2003) 9:1656–65. 12738718

[27] Nicholas NS Apollonio B Ramsay AG Tumor microenvironment (TME)-driven immune suppression in B cell malignancy. Biochim Biophys Acta - Mol Cell Res (2016) 1863:471–82. 10.1016/j.bbamcr.2015.11.003 26554850

[28] Rovida A Maccalli C Scarfò L Dellabona P Stamatopoulos K Ghia P Exploiting B Cell Receptor Stereotypy to design Tailored Immunotherapy in Chronic Lymphocytic Leukemia. Clin Cancer Res (2020). 10.1158/1078-0432.CCR-20-1632 33051305

[29] Khodadoust MS Olsson N Wagar LE Haabeth OAW Chen B Swaminathan K Antigen presentation profiling reveals recognition of lymphoma immunoglobulin neoantigens. Nature (2017) 543:723–7. 10.1038/nature21433 PMC5808925 28329770

[30] Scrivener S Goddard RV Kaminski ER Prentice AG Abnormal T-cell function in B-cell chronic lymphocytic leukaemia. Leuk Lymphoma (2003) 44:383–9. 10.1080/1042819021000029993 12688308

[31] Görgün G Holderried TAW Zahrieh D Neuberg D Gribben JG Chronic lymphocytic leukemia cells induce changes in gene expression of CD4 and CD8 T cells. J Clin Invest (2005) 115:1797–805. 10.1172/JCI24176 PMC1150284 15965501

[32] Nunes C Wong R Mason M Fegan C Man S Pepper C Expansion of a CD8 +PD-1 + replicative senescence phenotype in early stage CLL patients is associated with inverted CD4:CD8 ratios and disease progression. Clin Cancer Res (2012) 18:678–87. 10.1158/1078-0432.CCR-11-2630 22190592

[33] Hanna BS Roessner PM Yazdanparast H Colomer D Campo E Kugler S Control of chronic lymphocytic leukemia development by clonally-expanded CD8 + T-cells that undergo functional exhaustion in secondary lymphoid tissues. Leukemia (2019) 33:625–37. 10.1038/s41375-018-0250-6 30267008

[34] Buschle M Campana D Carding SR Richard C Victor Hoffbrand A Brenner MK Interferon γ inhibits apoptotic cell death in B cell chronic lymphocytic leukemia. J Exp Med (1993) 177:213–8. 10.1084/jem.177.1.213 PMC2190861 7678114

[35] Del Giudice I Chiaretti S Tavolaro S De Propris MS Maggio R Mancini F Spontaneous regression of chronic lymphocytic leukemia: Clinical and biologic features of 9 cases. Blood (2009) 114:638–46. 10.1182/blood-2008-12-196568 19387007

[36] Catakovic K Gassner FJ Ratswohl C Zaborsky N Rebhandl S Schubert M TIGIT expressing CD4+T cells represent a tumor-supportive T cell subset in chronic lymphocytic leukemia. Oncoimmunology (2018) 7(1):e1371399. 10.1080/2162402X.2017.1371399 PMC5739567 29296521

[37] Elston L Fegan C Hills R Hashimdeen SS Walsby E Henley P Increased frequency of CD4+PD-1+HLA-DR+ T cells is associated with disease progression in CLL. Br J Haematol (2020) 188:872–80. 10.1111/bjh.16260 31702049

[38] Gonzalez-Rodriguez AP Contesti J Huergo-Zapico L Lopez-Soto A Fernández-Guizn A Acebes-Huerta A Prognostic significance of CD8 and CD4 T cells in chronic lymphocytic leukemia. Leuk Lymphoma (2010) 51:1829–36. 10.3109/10428194.2010.503820 20846097

[39] Takeuchi Y Nishikawa H Roles of regulatory T cells in cancer immunity. Int Immunol (2016) 28:401–9. 10.1093/intimm/dxw025 PMC4986235 27160722

[40] Piper KP Karanth M McLarnon A Kalk E Khan N Murray J Chronic lymphocytic leukaemia cells drive the global CD4 + T cell repertoire towards a regulatory phenotype and leads to the accumulation of CD4 + forkhead box P3 + T cells. Clin Exp Immunol (2011) 166:154–63. 10.1111/j.1365-2249.2011.04466.x PMC3219890 21985361

[41] Lad D Hoeppli R Huang Q Garcia R Xu L Toze C Regulatory T-cells drive immune dysfunction in CLL. Leuk Lymphoma (2018) 59:486–9. 10.1080/10428194.2017.1330475 28573905

[42] Beyer M Classen S Endl E Kochanek M Weihrauch MR Debey-Pascher S Comparative approach to define increased regulatory T cells in different cancer subtypes by combined assessment of CD127 and FOXP3. Clin Dev Immunol (2011) 11:734036. 10.1155/2011/734036 PMC3166761 21904560

[43] Sakaguchi S Regulatory T Cells: Key Controllers of Immunologic Self-Tolerance. Cell (2000) 101:455–8. 10.1016/s0092-8674(00)80856-9 10850488

[44] Jak M Mous R Remmerswaal EBM Spijker R Jaspers A Yagüe A Enhanced formation and survival of CD4+ CD25hi Foxp3+ T-cells in chronic lymphocytic leukemia. Leuk Lymphoma (2009) 50:788–801. 10.1080/10428190902803677 19452318

[45] Roberts AW Davids MS Pagel JM Kahl BS Puvvada SD Gerecitano JF Targeting BCL2 with Venetoclax in Relapsed Chronic Lymphocytic Leukemia. N Engl J Med (2016) 374:311–22. 10.1056/NEJMoa1513257 PMC7107002 26639348

[46] Fischer K Al-Sawaf O Bahlo J Fink A-M Tandon M Dixon M Venetoclax and Obinutuzumab in Patients with CLL and Coexisting Conditions. N Engl J Med (2019) 380:2225–36. 10.1056/NEJMoa1815281 31166681

[47] Triulzi T Tagliabue E Balsari A Casalini P FOXP3 expression in tumor cells and implications for cancer progression. J Cell Physiol (2013) 228:30–5. 10.1002/jcp.24125 22674548

[48] Karanikas V Speletas M Zamanakou M Kalala F Loules G Kerenidi T Foxp3 expression in human cancer cells. J Transl Med (2008) 6:19. 10.1186/1479-5876-6-19 18430198 PMC2386447

[49] Nagai Y Lam L Greene MI Zhang H FOXP3 and Its Cofactors as Targets of Immunotherapies. Engineering (2019) 5:115–21. 10.1016/j.eng.2019.01.001

[50] Lozano T Gorraiz M Lasarte-Cía A Ruiz M Rabal O Oyarzabal J Blockage of FOXP3 transcription factor dimerization and FOXP3/AML1 interaction inhibits T regulatory cell activity: sequence optimization of a peptide inhibitor. Oncotarget (2017) 8:71709–24. 10.18632/oncotarget.17845 PMC5641083 29069740

[51] Yousefi M Movassaghpour AA Shamsasenjan K Ghalamfarsa G Sadreddini S Jadidi-Niaragh F The skewed balance between Tregs and Th17 in chronic lymphocytic leukemia. Futur Oncol (2015) 11:1567–82. 10.2217/fon.14.298 25963433

[52] Hus I Bojarska-Junak A Chocholska S Tomczak W Woś J Dmoszyńska A Th17/IL-17A might play a protective role in chronic lymphocytic leukemia immunity. PLoS One (2013) 8:1–8. 10.1371/journal.pone.0078091 PMC3815235 24223764

[53] Jadidi-Niaragh F Ghalamfarsa G Memarian A Asgarian-Omran H Razavi SM Sarrafnejad A Downregulation of IL-17-producing T cells is associated with regulatory T cell expansion and disease progression in chronic lymphocytic leukemia. Tumor Biol (2013) 34:929–40. 10.1007/s13277-012-0628-4 23269607

[54] Boćko D Kosmaczewska A Ciszak L Teodorowska R Frydecka I CD28 costimulatory molecule--expression, structure and function. Arch Immunol Ther Exp (Warsz) (2020) 50(3):169–77. 12098932

[55] Bonyhadi M Frohlich M Rasmussen A Ferrand C Grosmaire L Robinet E In Vitro Engagement of CD3 and CD28 Corrects T Cell Defects in Chronic Lymphocytic Leukemia. J Immunol (2005) 174:2366–75. 10.4049/jimmunol.174.4.2366 15699173

[56] Reichardt P Dornbach B Gunzer M APC, T cells, and the immune synapse. Curr Top Microbiol Immunol (2010) 340:229–49. 10.1007/978-3-642-03858-7_12 19960317

[57] Riches J C Ramsay A G G. Gribben J Immune Dysfunction in Chronic Lymphocytic Leukemia: The Role for Immunotherapy. Curr Pharm Des (2012) 18:3389–98. 10.2174/138161212801227023 22591385

[58] McClanahan F Hanna B Miller S Clear AJ Lichter P Gribben JG PD-L1 checkpoint blockade prevents immune dysfunction and leukemia development in a mouse model of chronic lymphocytic leukemia. Blood (2015) 126:203–11. 10.1182/blood-2015-01-622936 PMC4497961 25800048

[59] Gorgun G Ramsay AG Holderried TAW Zahrieh D Dieu R Liu F Eμ- TCL1 mice represent a model for immunotherapeutic reversal of chronic lymphocytic leukemia-induced T-cell dysfunction. Proc Natl Acad Sci U S A (2009) 106:6250–5. 10.1073/pnas.0901166106 PMC2669383 19332800

[60] Palma M Gentilcore G Heimersson K Mozaffari F Näsman-Glaser B Young E Mellstedt H. T cells in chronic lymphocytic leukemia display dysregulated expression of immune checkpoints and activation markers. Haematologica (2017) 102:562–72. 10.3324/haematol.2016.151100 PMC5394965 27927767

[61] Ramsay AG Clear AJ Fatah R Gribben JG Multiple inhibitory ligands induce impaired T-cell immunologic synapse function in chronic lymphocytic leukemia that can be blocked with lenalidomide: establishing a reversible immune evasion mechanism in human cancer. Blood (2012) 120(7):1412–21. 10.1182/blood-2012-02-411678 PMC3423779 22547582

[62] Darvin P Toor SM Sasidharan Nair V Elkord E Immune checkpoint inhibitors: recent progress and potential biomarkers. Exp Mol Med (2018) 50:165. 10.1038/s12276-018-0191-1 PMC6292890 30546008

[63] Hu B Jacobs R Ghosh N Checkpoint Inhibitors Hodgkin Lymphoma and Non-Hodgkin Lymphoma. Curr Hematol Malig Rep (2018) 13:543–54. 10.1007/s11899-018-0484-4 30338457

[64] Ding W LaPlant BR Call TG Parikh SA Leis JF He R Pembrolizumab in patients with CLL and Richter transformation or with relapsed CLL. Blood (2017) 129:3419–27. 10.1182/blood-2017-02-765685 PMC5492091 28424162

[65] Xu-Monette ZY Zhou J Young KH PD-1 expression and clinical PD-1 blockade in B-cell lymphomas. Blood (2018) 131:68–83. 10.1182/blood-2017-07-740993 29118007 PMC5755041

[66] Chanan-Khan A Porter CW Immunomodulating drugs for chronic lymphocytic leukaemia. Lancet Oncol (2006) 7:480–8. 10.1016/S1470-2045(06)70723-9 16750498

[67] Carballido E Veliz M Komrokji R Pinilla-Ibarz J Immunomodulatory drugs and active immunotherapy for chronic lymphocytic leukemia. Cancer Control (2012) 19(1):54–67. 10.1177/107327481201900106 22143062

[68] Ramsay AG Evans R Kiaii S Svensson L Hogg N Gribben JG Chronic lymphocytic leukemia cells induce defective LFA-1-directed T-cell motility by altering Rho GTPase signaling that is reversible with lenalidomide. Blood (2013) 121:2704–14. 10.1182/blood-2012-08-448332 PMC3617635 23325833

[69] Ramsay AG Johnson AJ Lee AM Gorgün G Le Dieu R Blum W Chronic lymphocytic leukemia T cells show impaired immunological synapse formation that can be reversed with an immunomodulating drug. J Clin Invest (2008) 118(7):2427–37. 10.1172/jci35017 PMC2423865 18551193

[70] Shanafelt TD Ramsay AG Zent CS Leis JF Tun HW Call TG Long-term repair of T-cell synapse activity in a phase II trial of chemoimmunotherapy followed by lenalidomide consolidation in previously untreated chronic lymphocytic leukemia (CLL). Blood (2013) 121:4137–41. 10.1182/blood-2012-12-470005 23493782

[71] Sakuishi K Apetoh L Sullivan JM Blazar BR Kuchroo VK Anderson AC Targeting Tim-3 and PD-1 pathways to reverse T cell exhaustion and restore anti-tumor immunity. J Exp Med (2010) 207:2187–94. 10.1084/jem.20100643 PMC2947065 20819927

[72] Blank C Kuball J Voelkl S Wiendl H Becker B Walter B Blockade of PD-L1 (B7-H1) augments human tumor-specific T cell responses in vitro. Int J Cancer (2006) 119:317–27. 10.1002/ijc.21775 16482562

[73] Klebanoff CA Rosenberg SA Restifo NP Prospects for gene-engineered T cell immunotherapy for solid cancers. Nat Med (2016) 22:26–36. 10.1038/nm.4015 26735408 PMC6295670

[74] Hanna BS Roessner PM Scheffold A Jebaraj BMC Demerdash Y Öztürk S PI3Kδ inhibition modulates regulatory and effector T-cell differentiation and function in chronic lymphocytic leukemia. Leukemia (2019) 33:1427–38. 10.1038/s41375-018-0318-3 30573773

[75] Norde WJ Hobo W Van Der Voort R Dolstra H Coinhibitory molecules in hematologic malignancies: Targets for therapeutic intervention. Blood (2012) 120:728–36. 10.1182/blood-2012-02-412510 22563087

[76] Virgin HW Wherry EJ Ahmed R Redefining Chronic Viral Infection. Cell (2009) 138:30–50. 10.1016/j.cell.2009.06.036 19596234

[77] Wherry EJ T cell exhaustion. Nat Immunol (2011) 12:492–9. 10.1038/ni.2035 21739672

[78] Zenz T Exhausting T cells in CLL. Blood (2013) 121:1485–6. 10.1182/blood-2013-01-475939 23449612

[79] Llaó Cid L Hanna BS Iskar M Roessner PM Öztürk S Lichter P CD8+ T-cells of CLL-bearing mice acquire a transcriptional program of T-cell activation and exhaustion. Leuk Lymphoma (2020) 61:351–6. 10.1080/10428194.2019.1660972 31519123

[80] Shapiro M Herishanu Y Ben-Zion-katz Dezorella N Sun C Kay S Lymphocyte activation gene 3: A novel therapeutic target in chronic lymphocytic leukemia. Haematologica (2017) 102:874–82. 10.3324/haematol.2016.148965 PMC5477606 28154084

[81] Vardi A Stamatopoulos K Hadzidimitriou A T cells in chronic lymphocytic leukemia: Can they fight? Oncotarget (2017) 8:99209–10. 10.18632/oncotarget.22277 PMC5725080 29245889

[82] Greaves M Clonal expansion in B-CLL: Fungal drivers or self-service. J Exp Med (2013) 210:1–3. 10.1084/jem.20122739 23319726 PMC3549708

[83] Os A Bürgler S Ribes AP Funderud A Wang D Thompson KM Chronic lymphocytic leukemia cells are activated and proliferate in response to specific T helper cells. Cell Rep (2013) 4:566–77. 10.1016/j.celrep.2013.07.011 23933259

[84] Minici C Gounari M Übelhart R Scarfò L Dühren-von Minden M Schneider D Distinct homotypic B-cell receptor interactions shape the outcome of chronic lymphocytic leukaemia. Nat Commun (2017) 8(1):15746. 10.1038/ncomms15746 28598442 PMC5472768

[85] Burger JA Tedeschi A Barr PM Robak T Owen C Ghia P Ibrutinib as Initial Therapy for Patients with Chronic Lymphocytic Leukemia. N Engl J Med (2015) 373:2425–37. 10.1056/NEJMoa1509388 PMC4722809 26639149

[86] Brown JR Byrd JC Coutre SE Benson DM Flinn IW Wagner-Johnston ND Idelalisib, an inhibitor of phosphatidylinositol 3-kinase p110δ, for relapsed/refractory chronic lymphocytic leukemia. Blood (2014) 123:3390–7. 10.1182/blood-2013-11-535047 PMC4123414 24615777

[87] Wiestner A BCR pathway inhibition as therapy for chronic lymphocytic leukemia and lymphoplasmacytic lymphoma. Hematology (2014) 2014:125–34. 10.1182/asheducation-2014.1.125 25696845

[88] Fais F Ghiotto F Hashimoto S Sellars B Valetto A Allen SL Chronic lymphocytic leukemia B cells express restricted sets of mutated and unmutated antigen receptors. J Clin Invest (1998) 102:1515–25. 10.1172/JCI3009 PMC509001 9788964

[89] Baliakas P Hadzidimitriou A Sutton LA Minga E Agathangelidis A Nichelatti M Clinical effect of stereotyped B-cell receptor immunoglobulins in chronic lymphocytic leukaemia: A retrospective multicentre study. Lancet Haematol (2014) 1:e74–84. 10.1016/S2352-3026(14)00005-2 27030157

[90] Damle RN Wasil T Fais F Ghiotto F Valetto A Allen SL Ig V Gene Mutation Status and CD38 Expression As Novel Prognostic Indicators in Chronic Lymphocytic Leukemia. Blood (1999) 94:1840–7. 10.1182/blood.v94.6.1840 10477712

[91] Hamblin TJ Davis Z Gardiner A Oscier DG Stevenson FK Unmutated Ig V(H) genes are associated with a more aggressive form of chronic lymphocytic leukemia. Blood (1999) 94:1848–54. 10.1182/blood.v94.6.1848 10477713

[92] Degan M Bomben R Bo MD Zucchetto A Nanni P Rupolo M Analysis of IgVH gene mutations in B cell chronic lymphocytic leukaemia according to antigen-driven selection identifies subgroups with different prognosis and usage of the canonical somatic hypermutation machinery. Br J Haematol (2004) 126:29–42. 10.1111/j.1365-2141.2004.04985.x 15198729

[93] Stamatopoulos K Agathangelidis A Rosenquist R Ghia P Antigen receptor stereotypy in chronic lymphocytic leukemia. Leukemia (2017) 31:282–91. 10.1038/leu.2016.322 27811850

[94] Wen T Mellstedt H Jondal M Presence of clonal T cell populations in chronic B lymphocytic leukemia and smoldering myeloma. J Exp Med (1990) 171:659–66. 10.1084/jem.171.3.659 PMC2187776 2137853

[95] Rezvany MR Jeddi-Tehrani M Österborg A Kimby E Wigzell H Mellstedt H Oligoclonal TCRBV gene usage in B-cell chronic lymphocytic leukemia: Major perturbations are preferentially seen within the CD4 T-cell subset. Blood (1999) 94:1063–9. 10.1182/blood.v94.3.1063.415a17_1063_1069 10419899

[96] Farace F Orlanducci F Dietrich PY Gaudin C Angevin E Courtier MH T cell repertoire in patients with B chronic lymphocytic leukemia: Evidence for multiple in vivo T cell clonal expansions. J Immunol (1994) 153:4281–90. 7930628

[97] Alatrakchi N Farace F Frau E Carde P Munck JN Triebel F T-cell clonal expansion in patients with B-cell lymphoproliferative disorders. J Immunother (1998) 21:363–70. 10.1097/00002371-199809000-00004 9789198

[98] Rezvany MR Jeddi-Tehrani M Wigzell H Österborg A Mellstedt H Leukemia-associated monoclonal and oligoclonal TCR-BV use in patients with B-cell chronic lymphocytic leukemia. Blood (2003) 101:1063–70. 10.1182/blood-2002-03-0746 12393705

[99] Hofbauer JP Heyder C Denk U Kocher T Holler C Trapin D Development of CLL in the TCL1 transgenic mouse model is associated with severe skewing of the T-cell compartment homologous to human CLL. Leukemia (2011) 25:1452–8. 10.1038/leu.2011.111 21606964

[100] Vardi A Agathangelidis A Stalika E Karypidou M Siorenta A Anagnostopoulos A Antigen Selection Shapes the T-cell Repertoire in Chronic Lymphocytic Leukemia. Clin Cancer Res (2016) 22:167–74. 10.1158/1078-0432.CCR-14-3017 26338994

[101] Vardi A Vlachonikola E Karypidou M Stalika E Bikos V Gemenetzi K Restrictions in the T-cell repertoire of chronic lymphocytic leukemia: High-throughput immunoprofiling supports selection by shared antigenic elements. Leukemia (2017) 31:1555–61. 10.1038/leu.2016.362 27904140

[102] Vardi A Vlachonikola E Papazoglou D Psomopoulos F Kotta K Ioannou N T cell dynamics in chronic lymphocytic leukemia under different treatment modalities. Clin Cancer Res (2020) 26:clincanres.3827.2019. 10.1158/1078-0432.CCR-19-3827 32616500

[103] Yin Q Sivina M Robins H Yusko E Vignali M O’Brien S Ibrutinib Therapy Increases T Cell Repertoire Diversity in Patients with Chronic Lymphocytic Leukemia. J Immunol (2017) 198:1740–7. 10.4049/jimmunol.1601190 PMC5296363 28077600

[104] Rajasagi M Shukla SA Fritsch EF Keskin DB DeLuca D Carmona E Systematic identification of personal tumor-specific neoantigens in chronic lymphocytic leukemia. Blood (2014) 124:453–62. 10.1182/blood-2014-04-567933 PMC4102716 24891321

[105] Fritsch EF Rajasagi M Ott PA Brusic V Hacohen N Wu CJ HLA-binding properties of tumor neoepitopes in humans. Cancer Immunol Res (2014) 2:522–9. 10.1158/2326-6066.CIR-13-0227 PMC4049249 24894089

[106] Kowalewski DJ Stevanovic S Rammensee HG Stickel JS Antileukemia T-cell responses in CLL – We don’t need no aberration. Oncoimmunology (2015) 4:1–3. 10.1080/2162402X.2015.1011527 PMC4485708 26140235

[107] Kowalewski DJ Schuster H Backert L Berlin C Kahn S Kanz L HLA ligandome analysis identifies the underlying specificities of spontaneous antileukemia immune responses in chronic lymphocytic leukemia (CLL). Proc Natl Acad Sci U S A (2015) 112:E116–75. 10.1073/pnas.1416389112 PMC4299203 25548167

[108] Khodadoust MS Olsson N Chen B Sworder B Shree T Liu CL B-cell lymphomas present immunoglobulin neoantigens. Blood (2019) 133:878–81. 10.1182/blood-2018-06-845156 PMC6384186 30545830

[109] Dunn GP Bruce AT Ikeda H Old LJ Schreiber RD Cancer immunoediting: From immunosurveillance to tumor escape. Nat Immunol (2002) 3:991–8. 10.1038/ni1102-991 12407406

[110] Parmar S Patel K Pinilla-Ibarz J Ibrutinib (Imbruvica): A novel targeted therapy for chronic lymphocytic leukemia(2014). Available at: https://www.ncbi.nlm.nih.gov/pmc/articles/PMC4103574/ (Accessed August 10, 2020). PMC4103574 25083126

[111] Mhibik M Wiestner A Sun C Harnessing the effects of BTKI on T cells for effective immunotherapy against CLL. Int J Mol Sci (2020) 21(1):68. 10.3390/ijms21010068 PMC6981459 31861854

[112] Dubovsky JA Beckwith KA Natarajan G Woyach JA Jaglowski S Zhong Y Ibrutinib is an irreversible molecular inhibitor of ITK driving a Th1-selective pressure in T lymphocytes. Blood (2013) 122:2539–49. 10.1182/blood-2013-06-507947 PMC3795457 23886836

[113] Kondo K Shaim H Thompson PA Burger JA Keating M Estrov Z Ibrutinib modulates the immunosuppressive CLL microenvironment through STAT3-mediated suppression of regulatory B-cell function and inhibition of the PD-1/PD-L1 pathway. Leukemia (2018) 32:960–70. 10.1038/leu.2017.304 PMC6128536 28972595

[114] Sagiv-Barfi I Kohrt HEK Czerwinski DK Ng PP Chang BY Levy R Therapeutic antitumor immunity by checkpoint blockade is enhanced by ibrutinib, an inhibitor of both BTK and ITK. Proc Natl Acad Sci U S A (2015) 112:E966–72. 10.1073/pnas.1500712112 PMC4352777 25730880

[115] Ujjani C Wang H Skarbnik A Trivedi N Ramzi P Khan N A phase 1 study of lenalidomide and ibrutinib in combination with rituximab in relapsed and refractory CLL. Blood Adv (2018) 2:762–8. 10.1182/bloodadvances.2017015263 PMC5894261 29610115

[116] Chellappa S Kushekhar K Munthe LA Tjønnfjord GE Aandahl EM Okkenhaug K The PI3K p110δ Isoform Inhibitor Idelalisib Preferentially Inhibits Human Regulatory T Cell Function. J Immunol (2019) 202:1397–405. 10.4049/jimmunol.1701703 30692213

[117] De Weerdt I Hofland T De Boer R Dobber JA Dubois J Van Nieuwenhuize D Distinct immune composition in lymph node and peripheral blood of CLL patients is reshaped during venetoclax treatment. Blood Adv (2019) 3:2642–52. 10.1182/bloodadvances.2019000360 PMC6737416 31506282

[118] Gandini S Massi D Mandalà M PD-L1 expression in cancer patients receiving anti PD-1/PD-L1 antibodies: A systematic review and meta-analysis. Crit Rev Oncol Hematol (2016) 100:88–98. 10.1016/j.critrevonc.2016.02.001 26895815

[119] Wierz M Pierson S Guyonnet L Viry E Lequeux A Oudin A Dual PD1/LAG3 immune checkpoint blockade limits tumor development in a murine model of chronic lymphocytic leukemia. Blood (2018) 131:1617–21. 10.1182/blood-2017-06-792267 PMC5887766 29439955

[120] Abken H Koehler P Schmidt P Hombach AA Hallek M Engineered T cells for the adoptive therapy of b-cell chronic lymphocytic leukaemia. Adv Hematol (2012) 2012:595060. 10.1155/2012/595060 21837241 PMC3152962

[121] Curran KJ Seinstra BA Nikhamin Y Yeh R Usachenko Y Van Leeuwen DG Enhancing antitumor efficacy of chimeric antigen receptor T cells through constitutive CD40L expression. Mol Ther (2015) 23:769–78. 10.1038/mt.2015.4 PMC4395796 25582824

[122] Gargett T Yu W Dotti G Yvon ES Christo SN Hayball JD GD2-specific CAR T Cells Undergo Potent Activation and Deletion Following Antigen Encounter but can be Protected from Activation-induced Cell Death by PD-1 Blockade. Mol Ther (2016) 24:1135–49. 10.1038/mt.2016.63 PMC4923328 27019998

[123] Torikai H Reik A Liu PQ Zhou Y Zhang L Maiti S A foundation for universal T-cell based immunotherapy: T cells engineered to express a CD19-specific chimeric-antigen-receptor and eliminate expression of endogenous TCR. Blood (2012) 119:5697–705. 10.1182/blood-2012-01-405365 PMC3382929 22535661

[124] Giannopoulos K Własiuk P Dmoszyńska A Roliński J Schmitt M Peptide vaccination induces profound changes in the immune system in patients with B-cell chronic lymphocytic leukemia. Folia Histochem Cytobiol (2011) 49:161–7. 10.5603/FHC.2011.0023 21526504

[125] Van Rooij N Van Buuren MM Philips D Velds A Toebes M Heemskerk B Tumor exome analysis reveals neoantigen-specific T-cell reactivity in an ipilimumab-responsive melanoma. J Clin Oncol (2013) 31(32):e439–e42. 10.1200/JCO.2012.47.7521 PMC3836220 24043743

